# Construction of a digital twin of chronic graft vs. host disease patients with standard of care

**DOI:** 10.1038/s41409-024-02324-0

**Published:** 2024-06-20

**Authors:** Gen Li, Yi-Bin Chen, Jonathan Peachey

**Affiliations:** 1 Phesi, East Lyme, CT USA; 2 Hematopoietic Cell Transplant & Cell Therapy Program, Massachusetts General Transplant Center: Bone Marrow Transplant Program, Boston, MA USA

**Keywords:** Phase 0 trials, Acute myeloid leukaemia

## Abstract

There is an unmet medical need for new clinical trials to evaluate novel therapies in chronic graft-versus-host disease (cGvHD). Disease rarity, ethical issues regarding placebo arms, time, and cost impede clinical trial conduct. Digital twin (DT) technology enables virtual clinical trial arm construction using historical data, circumventing these obstacles. We evaluated the feasibility of constructing a DT trial arm using a large database of real-world clinical trial data and performed an efficacy assessment of a standard-of-care (SOC) drug to examine agreement with literature data. We constructed a flGvHD DT cohort (cGvHD patients at first-line treatment) (2042 patients; 32 cohorts) using the Trial Accelerator™ Digital Twin platform and derived an SOC arm from this cohort (flGvHD DT SOC cohort) (438 patients; eight cohorts); we analyzed the efficacy of SOC (prednisone) (overall response rate (ORR)) at six months. Our analysis results are in agreement with literature: flGvHD DT: disease onset time: 7.58 months post-allogeneic hematopoietic cell transplantation; most used graft source: peripheral blood stem cells; flGvHD DT SOC: ORR at six months for prednisone: 52.7%. It is feasible to construct a DT cohort using existing clinical trial data; a DT SOC arm can potentially replace a control arm in clinical trials.

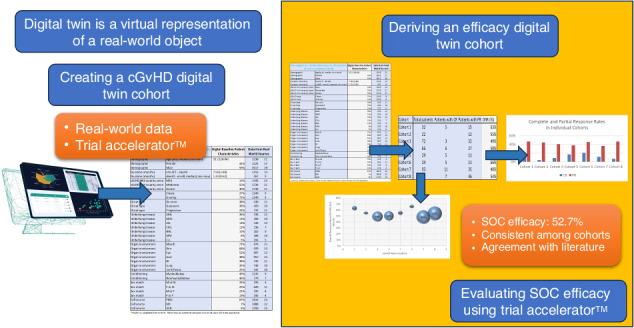

## Introduction

Chronic graft versus host disease (cGvHD) is a serious complication requiring systemic therapy that occurs in 30–50% of patients receiving allogeneic hematopoietic cell transplantation (HCT) [1]. Chronic GvHD typically appears >3 months after HCT and can affect almost any organ, most commonly the skin, eyes, and mouth [1]. The distinction between acute GvHD (aGVHD) and cGvHD is made purely based on clinical manifestations; “overlap syndrome” is defined in patients who exhibit symptoms of both aGvHD and cGvHD either at onset or with cGvHD developing in the context of prior aGvHD [2].

The standard-of-care (SOC) first-line treatment for cGvHD is systemic corticosteroids, with an ~40–60% overall response rate (ORR) [3]. However, long-term systemic steroids are associated with increased opportunistic infections and numerous other well-known toxicities and complications. Moreover, treatment resistance (steroid-refractory GvHD) or the use of a second-line agent to improve the quality of response or to be able to taper steroids more rapidly is fairly common. Currently, three FDA-approved targeted therapies are available for steroid-refractory cGvHD: ibrutinib, belumosudil, and ruxolitinib [4]. However, corticosteroids still remain as the first-line therapy. The “2020 treatment of cGvHD report” by the National Institutes of Health (NIH) cGvHD Consensus Project on Criteria for Clinical Trials (https://ncifrederick.cancer.gov/events/conferences/sites/default/files/inline-files/NIH_2020_CGvHD_conference_Introduction_PDF.pdf) emphasized the need to find viable alternatives to steroid therapy for cGvHD. However, to obtain meaningful results, large sample sizes are required, and patient recruitment places a considerable burden on time and financial resources [5]. In addition, randomizing patients to placebo arms often poses an ethical dilemma for investigators [6, 7] and makes patients reluctant to participate. Interestingly, the NIH cGvHD Consensus Project report also stressed that clinical trial designs other than large, randomized trials are required to test novel therapies. Therefore, there is an unmet need for novel clinical designs. In this context, advancements in digital technology and artificial intelligence (AI) have enabled investigators to rethink clinical trial design.

Digital Twin (DT) technology uses real-world data from clinical trials conducted worldwide and real-world evidence sources to create DT cohorts that can serve as placebo/standard-of-care (SOC)/contemporaneous control arms [7–9]. The US Food and Drug Administration (FDA) has recognized the immense potential of such technologies and has issued the “Artificial Intelligence/Machine Learning (AI/ML)-Based Software as a Medical Device (SaMD) Action Plan” (https://www.fda.gov/media/177030/download?attachment).

The strength of DT technology lies in the volume, diversity, and dynamic nature of the data used for DT construction; however, obtaining high-quality source data is a challenge. In this regard, Phesi, a clinical data science company, has compiled an extensive clinical development database and integrated it into its Trial Accelerator™ platform (includes data from >70 million patients; >485,000 cohorts; covers >4000 disease indications). The utility of this platform has been successfully demonstrated in two studies where DT SOC arms were constructed for efficacy evaluation in G12C non-small-cell lung cancer [8] and CAR-T cytokine release syndrome [7]. The aim is to use DT arms to eventually replace placebo/control arms to deliver time, cost, and ethical benefits.

In the present study, we created a DT arm for a clinical trial in cGvHD patients at first-line treatment using the Trial Accelerator™ DT platform; we used the DT cohort to elucidate the baseline characteristics of this digital arm; we further derived a SOC arm from this cohort and examined the efficacy of SOC (prednisone) in the cGvHD DT at six months after treatment initiation. It is hoped that this study will provide proof of concept for the application of DT technology for constructing DT arms in real-world clinical trials.

## Patients and methods

### First-line chronic GvHD digital twin cohort and analysis of baseline characteristics

A first-line GvHD Digital Twin (flGvHD DT) cohort was constructed using the Trial Accelerator™ clinical trial database. At the time of this analysis, this database included data from 61,224,369 patients in 232,909 cohorts. An AI algorithm was used to identify key patient attributes such as demographics, concomitant medication, comorbidities, disease status, and treatment outcomes. The AI algorithm mimics a manual search process to query and identify patient data aligned with our protocol design, including but not limited to patient inclusion and exclusion criteria, efficacy outcome measures, and treatment duration. The resulting patient data were subjected to quality analysis and verification steps by using human intelligence to ensure that all the details were considered.

### Eligibility criteria

*Inclusion criteria:* studies including patients with age ≥18 years, diagnosed with new-onset moderate or severe cGVHD as defined by 2014 NIH Consensus Development Project Criteria [10] or the NIH cGvHD response criteria [2], requiring systemic therapy, and history of one allogeneic HCT (any type of stem cell donor, any conditioning regimen and source of hematopoietic stem cells, any GVHD prophylaxis).

*Exclusion criterion:* studies with patients who received prior systemic therapy for cGvHD.

These criteria can be verified from the source documents (Supplementary Table S1). The patient population obtained after applying the inclusion and exclusion criteria was designated as the flGvHD DT cohort (Supplementary Fig. S1).

### flGvHD DT SOC cohort and analysis of efficacy of SOC

The following eligibility criteria were applied to the flGvHD DT cohort to further derive the flGvHD DT SOC (efficacy) cohort (Supplementary Fig. S1): flGvHD patients who received prednisone as first-line treatment, with or without an experimental regimen; efficacy measured as ORR at six months after starting therapy (defined as partial response (PR) + complete response (CR)) according to the 2014 NIH Consensus Development Project Criteria/NIH cGvHD response criteria [2, 10]. The outcome measure and treatment duration of the baseline patient population (flGvHD DT cohort) were tracked and aligned with the clinical trial design by manual inspection. The patient population obtained after applying these additional criteria was designated as the flGvHD DT SOC cohort (Supplementary Table S2); the ORR was compared across the cohorts. Overall survival (OS) data were also examined, but due to lack of availability of complete data (incomplete for 3/8 cohorts), OS was not included during the construction of the SOC DT.

### Statistical analysis

All analyses were performed by Phesi. All authors had access to all data. Data were summarized using descriptive statistics. Categorical variables are expressed as percentages and continuous variables as median values. A Chi-square test of independence was applied to evaluate whether there were statistically significant differences in the ORR and OS values among the included cohorts. The statistical significance was set at *p* < 0.05. Statistical analysis was performed using SAS version 9.4.

### Ethical considerations

The source data were obtained from published clinical trials and clinical studies (Supplementary Table S1). Therefore, ethical approval was not needed.

## Results

### flGvHD DT cohort

Of the 61,224,369 patients in 232,909 cohorts represented in the Trial Accelerator^TM^ database, 106,183 patients (770 cohorts) with any type of GvHD were identified. Of these, 17,769 adult patients (209 cohorts) with cGvHD were selected. Further, 2042 patients (32 cohorts) with cGvHD who received first-line treatment were finally selected and used to construct the flGvHD DT cohort (Supplementary Fig. S1). The patients were enrolled at centers distributed in >16 countries worldwide including the United States, Australia, China, Germany, France, Italy, Taiwan, Japan, South Korea, and Austria from 2004 to 2021 (references studies, Supplementary Table S1) (exception: one cohort from a 1997 study [11]).

### Baseline characteristics of flGvHD DT cohort

Table 1 shows the baseline characteristics of the flGvHD DT cohort. The median age of the DT cohort was 52.2 years, and the sex distribution was as follows: men, 59%, and women, 41%. The cGvHD onset type was either de novo or quiescent in many cases (38% and 39%, respectively), and appeared at a median of 7.58 months (range: 1–88 months) from time of HCT. More than half of the patients in the cohort exhibited moderate cGvHD (55%) as per the NIH severity scores, and the incidence of the overlap syndrome was high (overlap vs. classic GvHD, 72% vs. 27%). The most common underlying disease was acute myeloid leukemia (36%). The most common organs involved in cGvHD were the mouth and skin (71% and 68%, respectively). The graft source was peripheral blood stem cells in 89.6% of the included patients. The distribution of graft sources among patients in the cohorts is presented in Supplementary Table S3. Prophylaxis for GvHD was provided only to patients in Cohorts 3 and 4 (data not shown).

**Table 1:** The baseline characteristics of the chronic graft vs. host disease digital twin cohort at first-line treatment (flGvHD DT cohort).

Characteristic		Digital twin baseline patient characteristics—parameter values	Data from real-world sources used for constructing the DT	
			No. of cohorts	No. of patients
Demographic characteristic	Age (yrs), median (min-max)	52.2 (18–84)	21	1238
	Female	41%	17	1521
	Male	59%	28	2017
Duration (months, min-max)	allo-HCT - cGvHD	7.58 (1–88)	13	1152
	cGvHD - enrollment	1.33 (0–62)	9	467
cGvHD NIH severity score	Mild	16%	14	1443
	Moderate	55%	21	1734
	Severe	30%	21	1734
cGvHD type	Classic	27%	9	1249
	Overlap	72%	9	1249
Onset type	De novo	38%	11	430
	Quiescent	39%	10	409
	Progressive	24%	11	737
Underlying disease	AML	36%	12	334
	MDS	15%	10	284
	ALL	14%	12	334
	CML	13%	7	236
	NHL	10%	9	203
	MM	8%	10	286
	CLL	5%	5	191
Organ involvement	Mouth	71%	11	876
	Skin	68%	13	929
	Eye	52%	12	897
	Liver	48%	14	957
	GI	36%	11	743
	Lung	35%	10	746
	Joint/fascia	25%	10	541
Conditioning	Myeloablative	49%	9	1135
	Nonmyeloablative	46%	7	779
Graft sex match (host to recipient)	M to M	33%	4	195
	F to M	25%	11	826
	M to F	21%	4	195
	F to F	19%	4	195
Graft cell source	PBSC	87%	24	1816
	BM	7%	22	1888
	UCB	6%	15	1700

*AML* acute myeloid leukemia, *ALL* acute lymphoblastic leukemia, *BM* bone marrow, *CLL* chronic lymphocytic leukemia, *CML* chronic myeloid leukemia, *DT* digital twin, *F* female, *GI* gastrointestinal tract, *MDS* myelodysplastic syndrome, *MM* multiple myeloma, *M* male, *NHL* non-Hodgkin lymphoma, *PBSC* peripheral blood stem cells, *UCB* umbilical cord blood.

### flGvHD DT SOC cohort for efficacy analysis

The flGvHD DT cohort was used to construct a DT SOC arm (flGvHD DT SOC cohort) to evaluate the efficacy of prednisone (defined as the SOC treatment) as first-line therapy in these patients (Supplementary Fig. S1). Of the 2 042 patients from 32 cohorts in the flGvHD DT cohort, patients who were treated with prednisone as first-line treatment were included from studies that reported ORR (defined as CR + PR) measured at six months after initiation of treatment. Manual checks were performed to ensure that all the included studies used the 2014 NIH Consensus Development Project Criteria or the NIH cGVHD response criteria for efficacy evaluation. Due to heterogeneity in the studies with reference to the above parameters, only eight of the 32 cohorts satisfied the above criteria; 438 patients from eight cohorts were included. The patients were recruited from 195 investigator sites in 16 countries (Supplementary Table S2). Two cohorts (Cohorts 2 and 7) included one patient each with age <18 years.

**Fig. 1:**
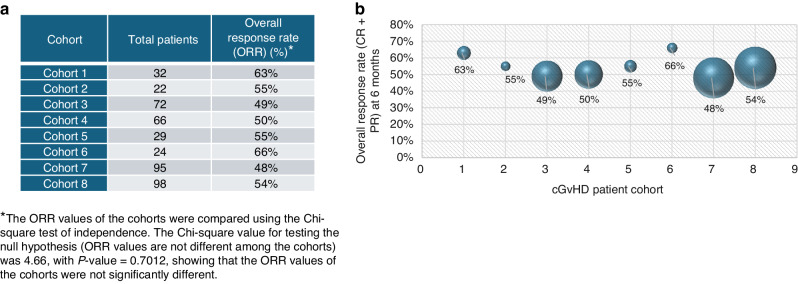
Summary of efficacy data (overall response rate defined as complete response (CR) + partial response (PR)) in the cohorts used to construct the flGvHD DT SOC cohort. **a** Table shows the details of the cohort size and efficacy data in the eight cohorts used to construct the DT. **b** Chart depicting the efficacy data of each cohort represented as spheres. The size of each sphere reflects the size of the relevant cohort, and the efficacy value is included for each sphere.

**Fig. 2:**
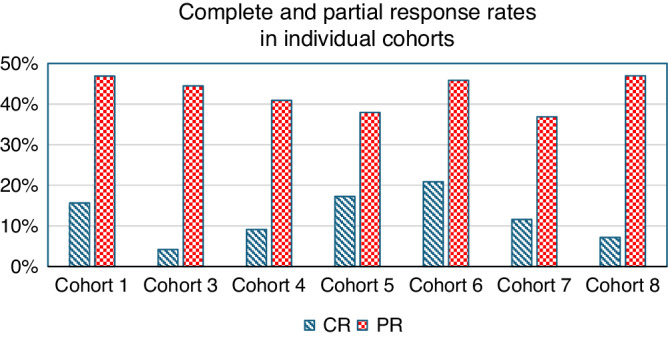
Complete and partial response rates in the individual cohorts included in the flGvHD DT SOC arm. CR complete response, PR partial response. *Note: complete data were unavailable for Cohort 2.

Our analysis showed that the ORR at six months (weighted based on the size of each cohort) after treatment initiation for prednisone was 52.7% (range: 48% [lowest in Cohort 7: 46 of 95 patients] to 66% [highest in Cohort 6: 16 of 24 patients]) (Fig. 1). The ORR was relatively stable across the eight cohorts, irrespective of the cohort size (range: 22 patients in cohort 2 to 98 in cohort 8). Chi-square analysis showed that there was no statistically significant difference among the ORR values of the eight cohorts (*χ*^2^ = 4.66; *p* = 0.70) (Fig. 1). Figure 2 shows the distribution of complete and partial response rates in the cohorts included in the flGvHD DT SOC arm (complete data unavailable for Cohort 2); the average PR was 43% (range: 37–47%), and the average CR was 12% (range: 4–21%).

In addition, the OS at 24 months was also examined for the five of eight cohorts with available data; the OS was 79% (range: 74% in Cohort 4 to 82% in Cohort 3) (Supplementary Table S4). Chi-square analysis showed that there was no statistically significant difference among cohorts with reference to OS (*χ*^2^ = 1.94; *p* = 0.75). However, due to the incompleteness of the dataset, OS was not considered in the construction of the flGvHD DT SOC cohort.

## Discussion

Combining digital data obtained from a patient pool comprised of various real-world data sources can predict the efficacy and possible adverse event profile of investigational drugs [7]. Recruiting and enrolling a large patient population for clinical trials is currently wrought with obstacles, and these issues are magnified in the case of rare diseases such as cGvHD. In addition, there is a trend towards redesigning of clinical trials to provide options other than large randomized studies, as exemplified by the opinions expressed in the “2020 treatment of cGvHD report” by the NIH cGvHD Consensus Project on Criteria for Clinical Trials (https://ncifrederick.cancer.gov/events/conferences/sites/default/files/inline-files/NIH_2020_CGvHD_conference_Introduction_PDF.pdf) and a call for alternative trial designs and need for trials without steroid treatment arms. In this study, we demonstrated the feasibility of creating a DT cohort of cGvHD patients in the first-line treatment setting (flGvHD DT cohort) using the Trial Accelerator™ DT platform, which includes a large database of patient data collected from various sources worldwide. This approach has been proven to objectively improve clinical development planning and protocol design optimization [7, 8]. Additionally, we also showed that this digital cohort can be used to construct a control treatment arm—the SOC arm—to validate the efficacy of standard treatment regimens; here we assessed the efficacy of prednisone as first-line treatment. Our findings using this simulation modeling approach are in agreement with and confirm findings from real-world clinical settings. This demonstrates that pre-existing and carefully collated data for a relevant and commonly applied therapy can be used to obtain meaningful results on drug efficacy. Such a digital arm can be used as a comparator arm for testing novel therapies in real-world clinical trials or for designing randomized studies with appropriate power and endpoints.

The baseline profile of the flGvHD DT cohort may be predictive of the patient profile obtained at the first-line treatment setting while applying the specific inclusion/exclusion criteria used here. The DT profile constructed herein suggests that patients present with cGvHD at a median of 7.5 months post transplantation. In agreement with the above, Arora et al. [12], in a prospective cohort study of 911 HCT recipients, reported that the time to onset of cGvHD was 7.4 months post transplantation [10]. Other studies have reported various post-transplantation onset times such as 162 days (5.4 months) [13], 153 days (5.1 months) [14], and 5.9 months (7.3 months for classic cGvHD, and 5.9 months for overlap type cGvHD) [15]. These time frames are well aligned with the 7.5-month timeframe derived from a very large and diversified dataset in the current study.

The ORR for first-line glucocorticoid therapy is reported to fall between 40 and 60% [3] and the efficacy analysis result for the flGvHD DT SOC is in agreement with the above, at 52.7% (measured at six months). An examination of the OS data (in cohorts with available source data) showed that the OS was 79% at 24 months after treatment initiation, with no statistically significant difference across cohorts (Supplementary Table S4). The above observations demonstrate that reliable and robust data can be obtained from diverse sources to construct DT arms and support the potential utility of DT SOC arms in real-world clinical trials.

While the analysis of individual patient data (IPD) associated with clinical trials has benefits, the resources, efforts, and inter-investigator cooperation required for such research will continue to limit the usage of IPD in many crucial areas of clinical research. In this regard, DTs can be used to obtain meaningful knowledge in a cost-effective manner. In addition, the regulatory approval of new drugs is based on aggregate patient data, rather than on IPD. Therefore, data from similar or identical studies employing the same agent can be combined with real-world patient data to accurately mimic placebo/comparator outcomes and create DT trial arms, which can then be utilized to detect early warning signs of trial results, safeguard patient safety, promote regulatory interaction, and improve submissions [7, 16]. As already mentioned, the FDA has been explicit about the value of such technologies and has issued the “Artificial Intelligence/Machine Learning (AI/ML)-Based Software as a Medical Device (SaMD) Action Plan”, which indicates that historical virtual control arms may be allowed in submission packages in the future. The method described here provides a reliable, consistent, and cost-effective option to construct historic control arms.

Despite its benefits, DT technology is associated with several challenges. First, as the strength of the technology lies in the size of the dataset used to construct the DT, the acquisition of large volumes of data is crucial; in this regard, data acquisition presents the most serious challenge [17]. In this study, the large size of the Trial Accelerator™ database allowed us to obtain an adequately large sample population for constructing the DTs. Second, as the data are sourced from numerous clinical studies conducted at diverse sites worldwide, data heterogeneity is a significant obstacle. In this study, only eight of the 32 cohorts could be included in the efficacy arm due to heterogeneity in the studies (outcome measures, treatment duration, and definition used for establishing ORR). Differences in the definition of the outcome measure (ORR) and treatment duration necessitated the exclusion of several cohorts during the construction of the efficacy DT. Additionally, data on failure-free survival (FFS), an increasingly important outcome measure in cGVHD studies, were not available for all the cohorts, and FFS was thus not used as an efficacy metric for the DT. It is important for clinical trial investigators to standardize the choice and reporting format of key parameters that can be used as modal values in AI-driven technologies. This will improve the accuracy and ease of development of DTs in the future. Third, as our study objective was to construct a DT of cGvHD patients at first-line treatment, we did not address the issue of “progressive onset” of cGvHD, even though the outcome in patients with progressive onset cGvHD is known to be significantly poor at first-line treatment. Fourth, the generalizability of the DT constructed here is limited to the population that falls strictly within the inclusion/exclusion criteria. It is thus important to validate this technology in various disease scenarios in GvHD. In addition, a small proportion of studies (4 of 18 in the baseline DT and 1 of 8 in the SOC cohort) are >10 years old. In our experience data shifts happen when quantifiable events occur, such as the introduction of a new treatment. As such events have not occurred, we did not feel the need to exclude these older studies. A statistical analysis of ORR data with and without Cohort 2 (older than 10 years) showed no statistical impact on the ORR results (data not shown).

In summary, AI-driven DT technology has the potential to significantly improve clinical trial design and implementation. In this study, we demonstrated the construction and application of a DT cohort in cGvHD for describing certain baseline clinical characteristics and showed that existing data can be used to validate that the SOC efficacy (as determined by the 6-month ORR) barrier that needs to be overcome by a new drug (to show superior efficacy compared to the SOC) is 52.7% in this population. The power of converting data into knowledge can be exemplified by the ease with which investigators can modify the inclusion/exclusion criteria in different scenarios to evaluate the effect of such changes on the baseline cohort profile and efficacy of the drug. The DT control arm could potentially function as an External Control Arm in a prospectively planned and implemented single-arm clinical trial, and solve certain ethical dilemmas surrounding placebo arms. It is hoped that this technology will gain regulatory approval in the future and find its place in the design and implementation of clinical trials.

### Supplementary information

Supplementary File 1

Supplementary File2

## Data Availability

All data used in the construction of the DT cohorts were obtained from published literature that has been cited here. Any further details may be obtained on reasonable request from info@phesi.com.

## Code availability

The AI algorithm used for performing search queries is a proprietary asset of Phesi, and thus the authors are unable to provide more details.
