# Supplementary material for: Construction of a digital twin of chronic graft vs. host disease patients with standard of care
Source: Bone Marrow Transplant. 2024 Jun 20;59(9):1280–5. doi: 10.1038/s41409-024-02324-0 (PMC11368802; doi:10.1038/s41409-024-02324-0)
Supplement: Supplementary file 1 [file 41409_2024_2324_MOESM1_ESM.pdf]

Supplementary Information

Supplementary Figure S1. Patient recruitment flow chart for constructing the flGvHD DT cohort and the flGvHD DT SOC cohort

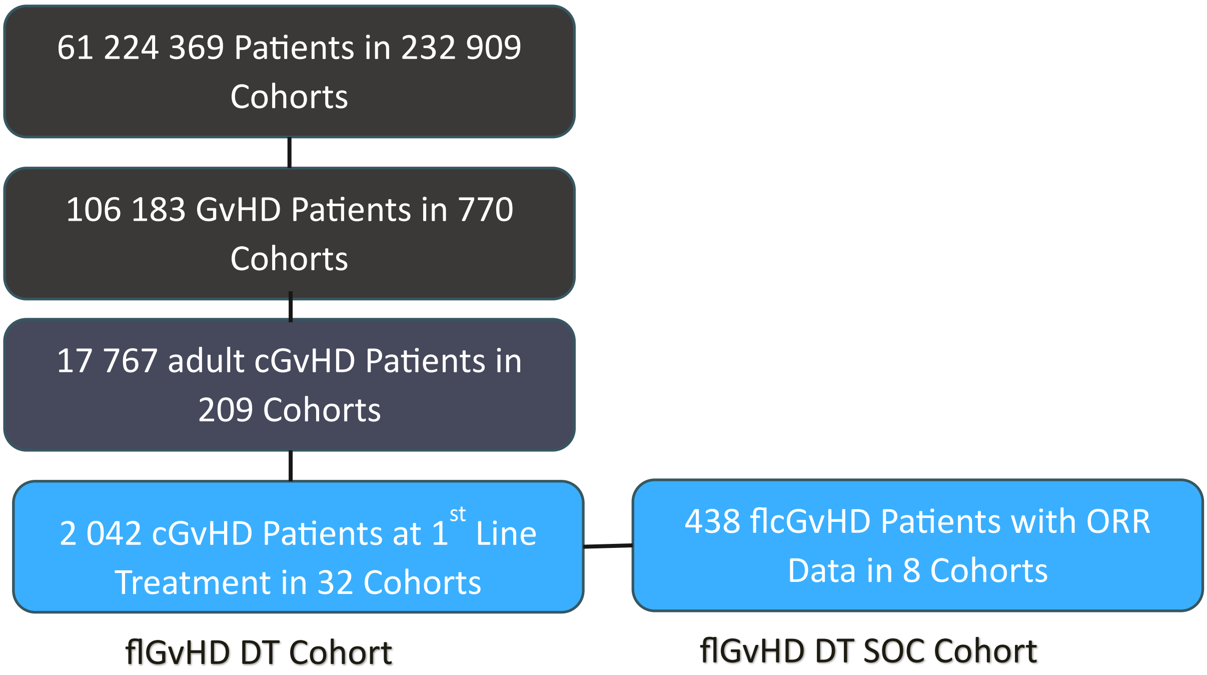

GvHD, graft vs. host disease; cGvHD, chronic GvHD; flcGvHD, cGvHD patients at first line treatment; ORR, overall response rate

Supplementary Table S2. Details of the studies reporting on cohorts used to construct the standard-of-care digital twin cohort (flGvHD DT SOC cohort) from the flGvHD DT cohort

	**Cohort 1**	**Cohort 2**	**Cohort 3**	**Cohort 4**	**Cohort 5**	**Cohort 6**	**Cohort 7**	**Cohort 8**
Lead investigator	Lazaryan A	Mitchell SA	Carpenter PA	Carpenter PA	Jagasia M	Jagasia M	Miklos DM	Miklos DM
Study duration	2014 to 2018	2005 to 2006	2010 to 2017	2010 to 2017	2011 to 2015	2011 to 2015	2017 to 2021	2017 to 2021
Randomized (Y/N)	N	N	Y	Y	Y	Y	Y	Y
Blinded (Y/N)	N	N	N	N	N	N	Y	Y
Number of arms	1	1	2	2	2	2	2	2
Definition of ORR	2014 NIH Consensus Development Project Criteria	NIH cGVHD response criteria			2014 NIH Consensus Development Project Criteria			
N	32	22	72	66	29	24	95	98
No. of countries	1	1	1	1	8	8	15	15
No. of study sites	17	3	44	44	30	30	102	102
Age: Median/Mean (range) or ± SD	59.5 (26 - 84)	33.5 (3 - 70)*	47.4 ± 16.1	50.9 ± 14.2	51.0 (23 - 72)	52.5 (24 - 68)	51 (13 - 72)*	56 (18 - 76)
Sex	M and F	M and F	M and F	M and F	M and F	M and F	M and F	M and F

*Cohort 2 and Cohort 7 included one participant each with age < 18 years. Data analysis revealed that there was no impact of the inclusion of these two participants on the results.

Y, yes; N, no; Other abbreviations are explained in Table 1

Supplementary Table S3. The distribution of graft source among the cGvHD patients in the eight cohorts that were used to construct the standard-of-care digital twin (flGvHD DT SOC)

Stem Cell Source	Cohort 1	Cohort 3	Cohort 4	Cohort 5	Cohort 6	Cohort 7	Cohort 8	Total
Cord blood	2	3	1	0	0	4	1	11
Bone marrow	1	5	5	4	2	8	8	33
Peripheral stem cells	35	64	60	25	22	84	90	380
Total	38	72	66	29	24	96	99	424

Note: Complete data were unavailable for Cohort 2, and thus this cohort has not been included in the table

Supplementary Table S4. The overall survival (OS) at 24 months after initiation of treatment of cGvHD patients in the eight cohorts that were used to construct the standard-of-care digital twin (flGvHD DT SOC)

Cohort	Total number of patients	Overall survival (24 months)	Average $\pm$ SEM and Chi-square test p-value
Cohort 1	32	77%	79 $\pm$ 0.3% $\chi^2 = 1.94$; $p = 0.75$
Cohort 2	22	NA	
Cohort 3	72	82%	
Cohort 4	66	74%	
Cohort 5	29	NA	
Cohort 6	24	NA	
Cohort 7	95	80%	
Cohort 8	98	80%	

NA: not available
